# Electron beam evaporation of superconductor-ferromagnet heterostructures

**DOI:** 10.1038/s41598-022-11828-y

**Published:** 2022-05-11

**Authors:** D. Bromley, A. J. Wright, L. A. H. Jones, J. E. N. Swallow, T. Beesley, R. Batty, R. S. Weatherup, V. R. Dhanak, L. O’Brien

**Affiliations:** 1 Department of Physics, University of Liverpool, Liverpool, L69 7ZE UK; 2 Department of Materials, University of Oxford, Parks Road, Oxford, OX1 3PH UK

**Keywords:** Superconducting properties and materials, Magnetic properties and materials

## Abstract

We report on the electronic and magnetic properties of superconductor-ferromagnet heterostructures fabricated by electron beam evaporation on to unheated thermally oxidised Si substrates. Polycrystalline Nb thin films (5 to 50 nm thick) were shown to possess reliably high superconducting critical temperatures ($T_{c}$), which correlate well with the residual resistivity ratio (RRR) of the film. These properties improved during *ex-situ* annealing, resulting in ${\Delta }T_{c}$ and ${\Delta }$RRR increases of up 2.2 K ($\sim$ 40% of the pre-annealed $T_{c}$) and 0.8 ($\sim$ 60% of the pre-annealed RRR) respectively. Nb/Pt/Co/Pt heterostructures showed substantial perpendicular anisotropy in the ultrathin limit (≤ 2.5 nm), even in the extreme limit of Pt(0.8 nm)/Co(1 nm)/Pt(0.6 nm). These results point to the use of electron beam evaporation as route to line-of-sight deposited, low-thickness, high quality Nb-based superspintronic multilayers.

## Introduction

Superconductor(*S*)-ferromagnet(*F*) heterostructures have revealed numerous phenomena such as spin-triplet production^1–4^ and supercurrents with tuneable macroscopic phase differences^5–7^ and continue to deepen our understanding of the interplay between these phases, particularly at interfaces^8,9^. When perpendicular magnetic anisotropy (PMA), which drives the *F* layer to point out-of-plane in equilibrium, is integrated within an *F* layer, e.g. via interfacial anisotropy, a candidate for cryogenic memory emerges based on superconducting spin electronics (superspintronics)^10^. Previous examples of scalable cryogenic memory cells have focused on multiple in-plane *F* layers^6,11–13^. Revising these geometries further, mixed anisotropy layers, where *F* layers are used with orthogonal anisotropies, may also be used to create magnetic inhomogeneity for studying the preservation of long-range triplet currents in *S-F-N-F* layers^14–16^, where *N* represents a normal metal. To this aim, work continues in the fundamental development of such *S*-*F* heterostructures^10,17–19^, particularly in simultaneously achieving sizeable PMA and reliable critical superconducting temperature, ${T}_{c}$, in multilayer films. Nb/Pt/Co layers represent a prototypical system where anisotropy may be tuned via the Pt/Co interface and inhomogeneous magnetic texture generated, at will^10,18,20^. Even in this system, however, work remains to develop heterostructures with both sizeable PMA and ${T}_{c}$ uninhibited by the large spin orbit coupling in Pt, *S–N* proximity effects^21^ and patterning effects^22^, particularly when integrated into devices.

Nb is often the superconducting material of choice as it benefits from fairly uncomplicated normal and superconducting phases, as well as relatively simple routes to thin film fabrication, the most prevalent being sputtered deposition^23–25^. While sputtering under ultra-high vacuum (UHV) offers high quality thin films and an easy route to building heterostructures, the technique is more challenging to integrate with, e.g., nanopatterning, due to the poor anisotropy of deposition angle for mask-based lithography and templating. It therefore remains beneficial for several device applications to explore alternative techniques when processing thin *S*-*F* heterostructures, e.g., considering potential 3D superspintronic devices. Alternative physical vapour deposition methods, such as electron beam evaporation (EBE), offer a potential approach; UHV EBE has previously been shown to generate smooth Nb thin films, with reliably high $T_{c}$^26,27^. In particular, UHV EBE offers highly anisotropic, line-of-sight deposition, which is ideally suited towards templated lithography, positive resist (lift-off) patterning, glancing angle deposition methods and 3D scaffolded growths.

Sputtering is equally ubiquitous as a method of deposition for thin film PMA magnetic layers, owing to, for example, the favourable growth kinetics for thin films and relative ease to which stoichiometric compounds may be grown^28–30^. Nonetheless, the ability to fabricate an all-EBE *S*-*F* heterostructure would be appealing as a route to low-profile nano- and 3D-patterning compatible superspintronic devices. Previous works have successfully demonstrated EBE grown PMA layers, although these rely on techniques, such as, multilayers^31^, superlattices^32^, and epitaxial alloys^33^. Simple trilayer structures, such as Pt/Co/Pt, would offer greater simplicity, however, these require compatible substrate wetting and growth morphology when fabricated via EBE, which often limits applicability. Therefore, while EBE is a common approach for growth of many metallic thin films, to-date, it has not been readily explored for superspintronic heterostructure growth, particularly in the very thin limit (Nb thickness $t_{Nb} <$ 10 nm).

In this paper, we systematically investigate EBE as a facile technique for the fabrication of low thickness *S-F* heterostructures, for use in superspintronic applications. First, we examine the electronic, structural and superconducting properties of thin EBE-grown Nb layers with thicknesses $t_{Nb}$ in the range 5–50 nm, capped with either MgO(3) or AlO_x_(3) oxidation barriers (thicknesses in nm in parentheses). These isolated Nb thin films are shown to have $T_{c}$ > 4 K, even in the thinnest ($t_{Nb}$ = 5 nm) films measured. We then anneal *ex-situ* and under high vacuum conditions at temperatures ranging 300–600 $^\circ$C in order to optimise $T_{c}$. Following this, we explore *S-F* heterostructures using ultrathin Pt/Co/Pt as an *F* layer, with Pt and Co thicknesses chosen to generate significant PMA, illustrating EBE-grown Nb to be a suitable seed layer for achieving PMA at room and low temperatures. As EBE is amenable to thin films and line-of-sight deposition, this affords the opportunity to better study interfaces, tunnelling effects and patterned devices (including glancing angle deposition coating for 3D superspintronics). Despite the prevalence of sputtered PMA heterostructures and superconducting spintronic device, here we demonstrate that EBE is a useful technique in generating low-thickness, high quality superspintronic multilayers.

## Results and discussion

### Properties of thin film Nb of varying thicknesses

**Figure 1:**
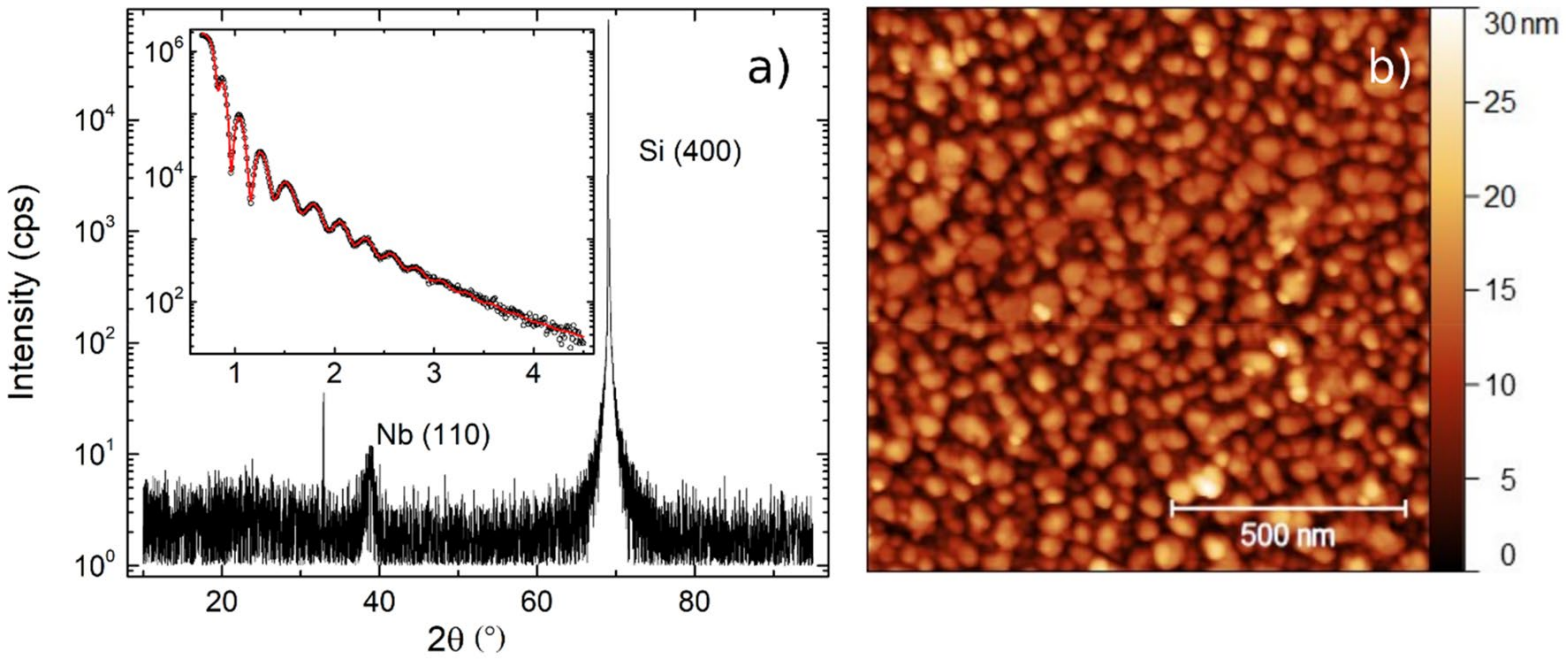
(**a**) XRD spectra of a thin film sample, with Nb (110) and Si substrate (400) peaks identified. Inset shows an XRR scan for the same sample. The solid red line is a fit to the data. (**b**) AFM image of the same Nb(30)/AlO_x_(3) thin film.

First, to understand the electronic, structural and superconducting properties of thin Nb layers, we examine SiO_x_/Nb($$t_{Nb} )$$/MgO(3) and SiO_x_/Nb($$t_{{{{Nb}}}} )$$/AlO_x_(3) bilayers (AlO_x_ is formed through in-air passivation of an Al layer). A typical x-ray diffraction (XRD) $$\theta$$-$$2\theta$$ scan of an EBE grown Nb layer [SiO_x_/Nb(30)/AlO_x_(3)] is shown in Fig. 1a. The single broad, low amplitude peak at 2θ $$=$$ 38.3° indicates a weakly (110) textured, polycrystalline Nb film. The relevant x-ray reflectivity (XRR) scan is shown in the inset of Fig. 1a, where black open circles show the collected data and the red line shows a reflectivity curve for a refined fit profile^34^ corresponding to Nb(30)/AlO_x_(3) with an interfacial roughness of 1.7 nm. To support these data an AFM image is shown in Fig. 1b. There, a polycrystalline film is seen with a calculated lateral grain size of ~ 20 nm. Prior work on room temperature EBE grown Nb thin films ($$t_{Nb} =$$ 10–100 nm) has found low roughness (several nm RMS), weakly textured, polycrystalline films of ~ 10 nm grain size, when deposited on Si^26^. Taken in conjunction, our structural characterisation further supports these observations with similar morphology and roughness of the Nb layers displayed here, even in the thin limit (5–50 nm).

**Figure 2:**
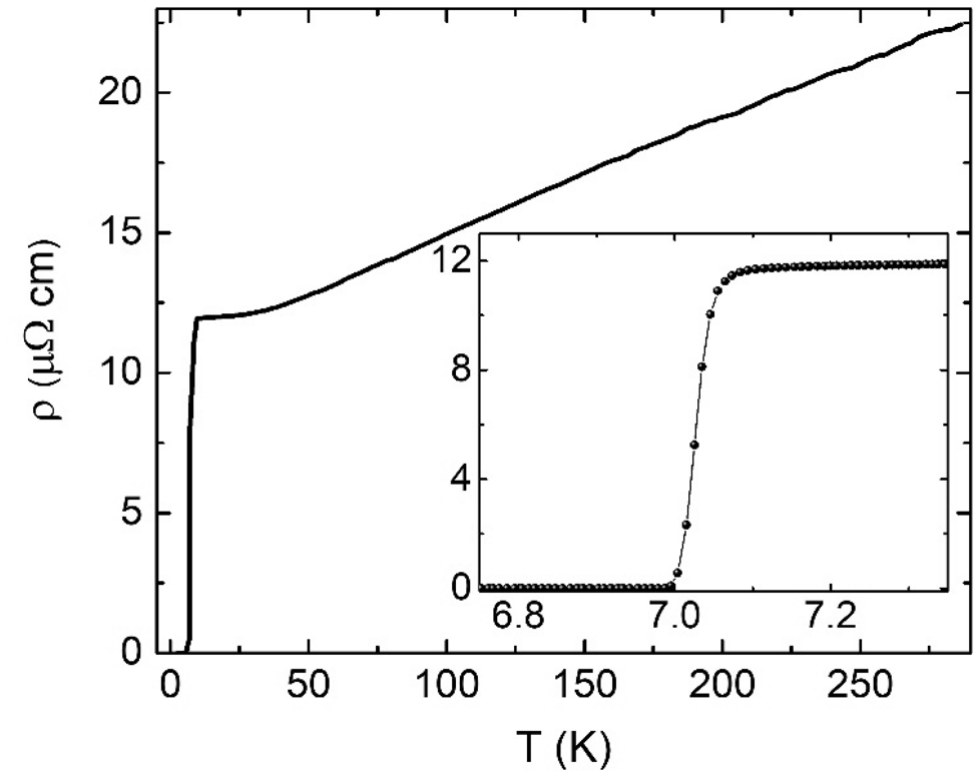
Resistivity, $\rho$, as a function of temperature, $T$, for a Nb(20)/MgO(3) sample measured using the van der Pauw method. The inset illustrates the superconducting transition in greater detail. $T_{c}$ was found to be 7.03 K. All $T_{c}$ values reported in this study are determined using a 50% resistance criterion ^37^.

**Figure 3:**
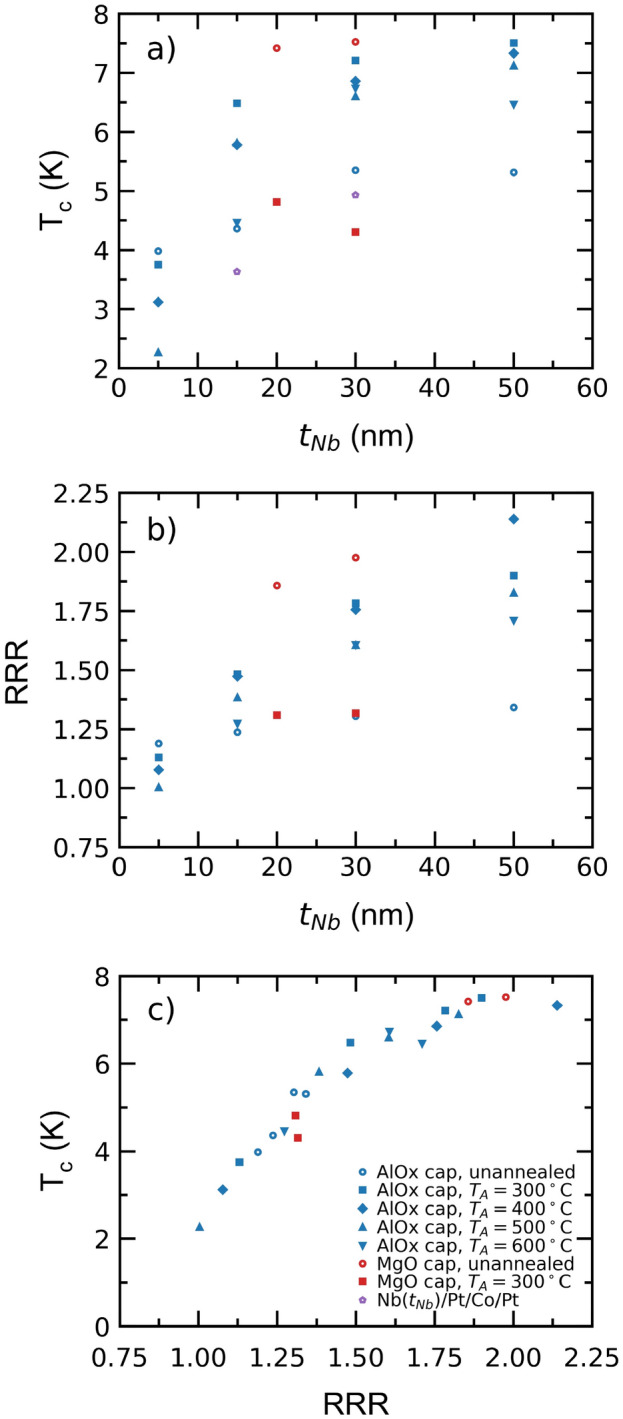
Data from transport measurements of Nb layers. Error bars are smaller than the symbol size. (**a**) $T_{c}$ as a function of Nb thickness, $t_{Nb}$. Examples of $T_{c}$ for *S-F* heterostructures are shown in purple; (**b**) RRR as a function of $t_{Nb}$. (**c**) $T_{c}$ as a function of RRR for all samples seen in (a) and (b).

We next report the transport properties of thin EBE Nb layers, ranging from $t_{Nb} =$ 5–50 nm. A representative plot of film resistivity, $\rho \left( T \right)$, for the sample series is shown in Fig. 2, taken from a Nb(20)/MgO(3) sample. Closer inspection of the superconducting phase change region (see Fig. 2 inset) reveals a narrow step like transition (< 70 mK wide). Comparable features are observed in all unannealed samples, with little variation in the width or functional form of the $\rho \left( T \right)$ superconducting transition. The superconducting transition temperature, $T_{c}$, and residual resistance ratio (RRR) [defined as $\rho \left( {T = 300 K} \right)/\rho \left( {T = 10 K} \right)$] are extracted and shown as a function of $t_{Nb}$ in Fig. 3a,b, respectively. In all panels, data is shown for samples with MgO (red data) and AlO_x_ (blue data) caps. In Fig. 3a we see that the superconducting transition is remarkably robust in these thin unannealed Nb layers (hollow symbols), even down to $t_{Nb} =$ 5 nm, with $T_{c}$ > 4 K in all cases. For a given sample series (for example, Nb films capped with Al and annealed at 300 °C constitute a sample series) there is a broad trend which shows $T_{c}$ to increase on increasing $t_{Nb}$. This is also found to be the case when comparing RRR to $t_{Nb}$ and has been observed in previous studies^25–27,35,36^. For completeness, also shown in Fig. 3a are the resulting $T_{c}$ values for the S/F heterostructures discussed later in this work [Nb($t_{Nb}$)/Pt(2)/Co(0.8)/Pt(1.5)]. Proximity-induced $T_{c}$ suppression is clear in these samples, with $\sim$ 1 K reduction in $T_{c}$, when compared with the lone Nb films.

Further inspection of Fig. 3b reveals low values of RRR ~ 1–2 throughout. Such low values are typical^26,38^ of thin polycrystalline films when momentum scattering from grain boundaries, which are relatively frequent due to the small grain size in our samples (see Fig. 1b), and/or surface scattering as $t_{Nb}$ approaches the electron mean free path, dominate. We may compare the trends of Fig. 3 with those found in epitaxial thin film samples. Thick ($t_{Nb} =$ 400 to 600 nm), (110) oriented Nb films epitaxially grown on MgO and SrTiO_3_ show ‘mesh-like’ crystal growth, with RRR > 100 and $T_{c} =$ 9.2 and 8.7 K, respectively^39^. NaCl substrates allow for (001) oriented epitaxial growth, with ultrathin ($t_{Nb} =$ 4 to 100 nm) films displaying RRR between 1 and 5 and $T_{c}$ ranging between 2 and 8.5 K^27^. Similarly, epitaxial thin films grown on (0001) Al_2_O_3_ display RRR = 6, with $T_{c} =$ 9.1 K for $t_{Nb} >$ 40 nm, down to RRR ~ 1.5 and $T_{c} =$ 6.5 K at $t_{Nb} =$ 10 nm ^25^. In thicker films, regardless of choice of Al_2_O_3_ orientation, RRR has been generally found to exceed 90, with $T_{c}$ approaching bulk values, $T_{c} \sim$ 9.2 K^40^. Clearly, depending on substrate choice and growth parameters, a wide variation in transport properties can be displayed, however, we naturally see a reduced RRR and $T_{c}$ across all thicknesses tested for our polycrystalline films. Despite the generally larger RRR and $T_{c}$, the trends in Fig. 3 nevertheless match the low thickness ($t_{Nb} \le 15$ nm) epitaxial system behaviour closely, particularly, in Jiang et al.^27^, potentially pointing to a dominance of finite size effects, such as weak localisation, lifetime broadening and surface scattering. While these $T_{c}$ values are reduced compared with epitaxial systems, they show clear consistency with polycrystalline films and growth on Si substrates. There, structural disorder and finite size effects are consistently found to suppress RRR and $T_{c}$^26,41–43^$,$ giving quantitatively similar dependence on $t_{Nb}$ as seen here.

In Fig. 3c we compare $T_{c}$ with the implicit variation in RRR across the sample series and a clear relation, for both annealed and unannealed samples, becomes apparent. Song et al. demonstrated that $T_{c}$ is contingent on the ratio of the electron–defect scattering rate and the electron–phonon coupling parameter in Nb films, of which RRR can be used as an unambiguous proxy^44^. This finding is further substantiated by the clear monotonic dependence in our data.

**Figure 4:**
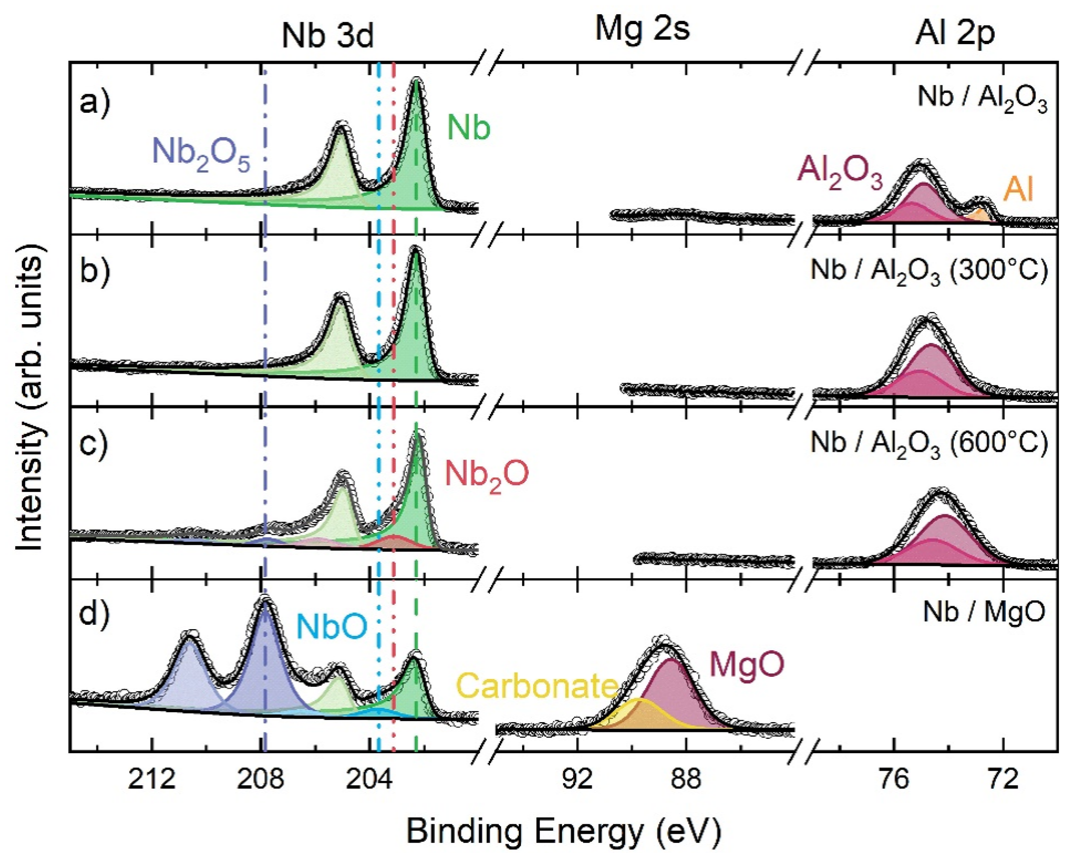
XPS characterisation illustrating the Nb 3d, Al 2p core levels for Al_2_O_3_ capped Nb (**a**) unannealed, annealed at (**b**) 300 °C and (**c**) 600 °C and (**d**) the Nb 3d and Mg 2 s core levels for MgO capped Nb. The carbonate peak observed at roughly 90 eV can be explained simply by atmospheric contaminants. All Nb films are 30 nm thick. The binding energies for all elements and compounds seen within this figure are corroborated by those ascertained in previous works ^50–58^.

We next examine the effect of annealing temperature, $T_{A}$, on the Nb thin films (full symbols). In Fig. 3a,b it is evident for AlO_x_ capped samples, where $t_{Nb}$ $\ge$ 15 nm, both $T_{c}$ and RRR increase on annealing. However, there is a clear non-monotonic dependence on $T_{A}$, with annealing at $T_{A} =$ 300 °C producing larger increases in $T_{c}$ and RRR, compared with $T_{A} =$ 600 °C. The effect of annealing is clarified by the X-ray photoelectron spectroscopy (XPS) data shown in Fig. 4a–c for Nb/AlO_x_ samples. In the unannealed Nb, a peak attributable to metallic Al is visible at 73 eV but disappears for the annealed samples, indicating the oxidation of residual Al metal at the Nb/AlO_x_ interface, i.e. the removal of a source of proximity-induced $T_{c}$ suppression^45^. However, annealing at higher temperatures ($T_{A} =$ 600 °C) produces Nb_2_O peaks in the XPS data, a clear indicator that oxygen has penetrated into the Nb film (the probing depth of our XPS measurement is ~ 10 nm, of which 3 nm will be capping material). This is corroborated by the lower RRR values (i.e. higher impurity density) for samples annealed at $T_{A} =$ 600 °C, compared with $T_{A} =$ 300 °C.

We interpret the increase in both RRR and $T_{c}$ under low temperature annealing ($T_{A} <$ 400 °C) as the removal of defects (point defects, vacancies, impurities) in the polycrystal films. Examining AFM measurements of annealed Nb films, we see a modest increase in grain size from 20 to ~ 24 nm for $T_{A} =$ 600 °C, confirming only marginal grain size changes even for the highest temperatures accessed. This observation is consistent with other post-growth annealing experiments of both Nb films ($t_{Nb} =$ 300 nm) and bulk experiments, where changes in microstructure are found only above $T_{A} >$ 500 °C^46^ and initiation of recrystallisation on macroscopic lengths is only observed above 900 °C^47^. This observation is also in agreement with the observed annealing dependence of transport measurements in Fig. 3: Above $T_{A} =$ 300 °C, little further improvement in RRR is seen, which suggests RRR (and $T_{C}$) become limited by finite size effects and/or grain boundary and surface scattering between 300 and 500 °C^42,43^. Indeed, a similar finding was observed in a previous study^46^ of sputtered Nb thin films in which *ex-situ* annealing, under comparable pressures and temperatures to those seen here, was performed. Using a Mayadas-Shatzkes resistivity model for polycrystalline thin film metals^48^, Lacquaniti et al*.* demonstrated reductions in RRR to be the result of oxygen diffusion in to the Nb grains^46^, as appears to be the case here. Looking to the $t_{Nb}$ = 5 nm samples, $T_{c}$ and RRR consistently decrease with increasing anneal temperature, which would suggest oxidation of the Nb grains throughout the thickness of the $t_{Nb}$ = 5 nm film, again consistent with both XPS data and Ref.^46^.

Finally we turn to the samples capped with MgO. Left unannealed, these samples have larger $$T_{c}$$ and RRR values than their Al-capped counterparts, despite there being greater amounts of NbO_x_ present near the surface (see Fig. 4d). The presence of Nb_2_O_5_ in Fig. 4d is naturally associated with passive oxidation either via O migration through, or directly from, the MgO layer (the high source purity and very low O partial pressure during deposition precludes substantial bulk contamination). From O tracer diffusion experiments^49^, the O diffusion length into Nb at room temperature, $$\lambda (T =$$ 30 °C $$)\sim$$ 0.5 nm, thus this Nb_2_O_5_ layer is likely limited by diffusion kinetics to the very near Nb/MgO interface, consistent with the surface (< 10 nm) sensitive XPS probe. With oxidation localised to the near-surface, this has little impact on the overall transport properties of the bulk film; RRR and $$T_{c}$$ remain high. However, on annealing to $$T_{A} =$$ 300 °C, O diffusion length increases rapidly, $$\lambda (T =$$ 300 °C $$) >$$ 500 nm, i.e $$. \lambda \gg t_{Nb}$$ and any interfacial excess O penetrates throughout the Nb film, rapidly reducing $$T_{c}$$ and RRR.

We note, comparison between Nb/MgO and Nb/AlOx data shows differences in the C1s spectra of the different capped samples. It is evident from the C1s signal that there is a notable amount of C intensity at ~ 289 eV in comparison to the AlOx spectra, which is attributed to a carbonate, the absence of which in the AlOx capped samples may arise from minor differences in growth environment, sample processing or atmospheric contamination between growth and XPS measurement.

### Magnetic anisotropy in EBE deposited ultra-thin *S-F* heterostructures

We next report on the magnetic and magneto-transport properties of thin EBE *S-F* devices with a structure of SiO_x_/Nb(15)/Pt($t_{Pt,b}$)/Co($t_{Co}$)/Pt($t_{Pt,t}$)/Cu(0,5)/MgO(3), in which Pt/Co/Pt is used as an *F* layer. Here, $t_{Pt,b}$ ($t_{Pt,t}$) is varied between 0.8 and 2.5 nm (0.6–1.5 nm) and $t_{Co}$ is either 0.8 or 1 nm. Heterostructures were grown with and without the inclusion of a 5 nm Cu layer on top of the *S-F* layers, i.e. Cu(0,5). The comparison of these differing structures allowed us to test the possible presence and influence of oxidation of the Pt/Co/Pt ferromagnet under the MgO capping layer and provide a *N* decoupling layer for future growth of mixed anisotropy *S-F-N-F* triplet valves. We detected no difference between the magnetic reversal of the two structures so do not discern further between the two structures in this work.

**Figure 5:**
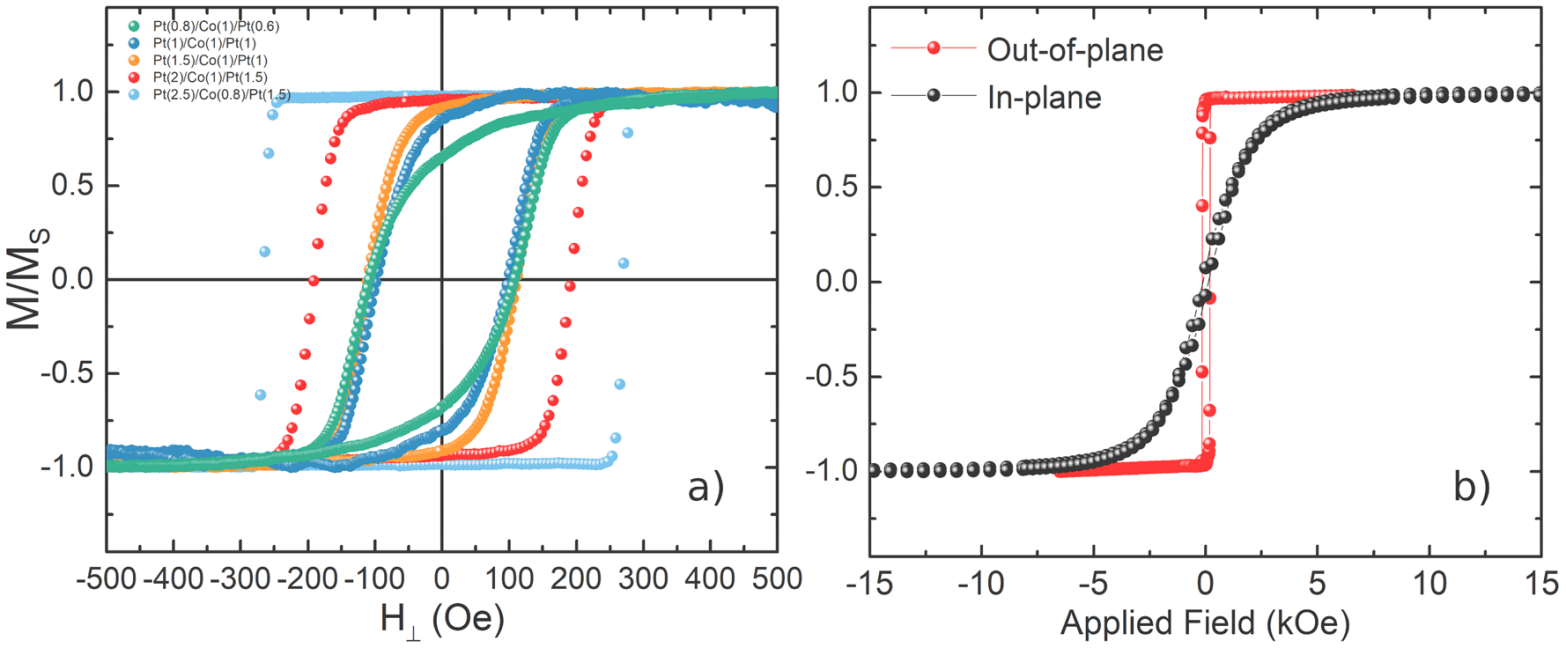
Room temperature magnetometry. All samples shown have a structure of Nb(15)/Pt/Co/Pt/Cu(0,5)/MgO(3). Fields are swept at 200 Oe/s. (**a**) Polar MOKE hysteresis loops (red and light blue markers indicate those without Cu i.e. 0 nm), where the Pt/Co/Pt trilayers are described in the legend. All thickness values are in nm. (**b**) Representative in-plane and out-of-plane hysteresis loops taken via SQUID vibrating sample magnetometry of the Nb(15)/Pt(2)/Co(0.8)/Pt(1.5)/MgO(3) structure. This sample has a $T_{c} \approx$ 3.64 K.

Normalised out-of-plane hysteresis curves $M_{Z} \left( H \right)/M_{s}$, obtained via polar magneto-optical Kerr effect (MOKE) microscopy, are shown in Fig. 5 for different Nb(15)/*F*/Cu(0,5)/MgO(3) samples (*F* = Pt/Co/Pt). For comparison, samples without a Nb underlayer were also grown: SiO_x_/Pt(2)/Co($t_{Co}$)/Pt(1), where $t_{Co} =$ 1.5, 2, and 2.5 nm. The *S-F* films show out-of-plane remanence, up to 98% for Nb(15)/Pt(2.5)/Co(0.8)/Pt(1.5) (see Fig. 5). This is in stark contrast to the unbuffered Pt/Co/Pt layers, which all displayed in-plane remanence, with an upper estimate for the effective anisotropy $K_{eff} \sim$ − 7$\times$ 10^5^ J/m^3^. (The negative sign here indicates a hard axis normal to the film plane.) Naturally, for the Nb buffered samples, this indicates substantial PMA in the *S-F* heterostructures (1.2 $\times$ 10^6^ J/m^3^ is required to overcome shape anisotropy); a finding that is significant given the Pt layer underneath the Co is ultra-thin (0.8 $< t_{Pt,b} <$ 2.5 nm). Evidencing this further, Fig. 5b shows in plane and out-of-plane hysteresis loops obtained via SQUID magnetometry, for a Nb(15)/Pt(2)/Co(0.8)/Pt(1.5)/MgO(3) structure. The substantial PMA is evident here, with a clear easy axis perpendicular to the film plane and anisotropy field of $H_{K} \sim$ 4 kOe. Hybridisation at the Pt/Co interface is known to induce substantial PMA in the Co layer, however, this requires the formation of a well-ordered Pt/Co interface and hence is typically weak in thin, un-buffered and unannealed (EBE) films, which typically exhibit poor wetting, and hence island-like growth of Pt on Si. This surface deposition energy is a common limiting factor when considering Pt growth on Si and is typically mitigated by the use of a refractory metal buffer layer, e.g. Ta^59^, to seed layer-by-layer growth. Here we see that the Nb(15) underlayer acts as an effective seed to Pt growth and so the combined stack provides an effective source of PMA, e.g., for mixed anisotropy spin valves, even at the low $t_{Pt,b}$ values required for superspintronic applications. To probe the Pt/Co/Pt interfaces, we also measure uncapped Nb(15)/Pt(2), Nb(15)/Pt(2)/Co(0.8) and Nb(15)/Pt(2)/Co(0.8)/Pt(1.5) films via AFM. These show an RMS roughness of 0.5 nm for both the Pt/Co and Co/Pt interfaces, with sub-10 nm grain size for both the lower Pt and Co layers, emphasising smooth, low roughness and small grain Pt films grown on the Nb(15) buffer.

From Fig. 5 we gain a qualitative understanding of how the normalised remanent magnetisation $M_{R} /M_{s}$, (defined as $M\left( {H = 0} \right)/M_{s}$) and coercive field, $H_{c}$, evolve as $t_{Pt,t}$ and $t_{Pt,b}$ are varied. Examining $M_{R} /M_{s}$, we see that, as we reduce the Pt thickness on either side of the Co, $M_{R} /M_{s}$ decreases. Previous studies have shown PMA strength to overtly depend on the $t_{Pt}$ at the bottom Pt/Co interface^60,61^. In particular, this arises due to the increased interfacial roughness that accompanies a reduction in $t_{Pt,b}$, as well as the finite thickness required to form a complete Pt/Co interface^60,61^. The thinnest Pt layers ($t_{Pt,b} <$ 1 nm) are comparable to this interface inhomogeneity and, in this thin limit (which we explore in an overall effort to minimise the deleterious effects of Pt on supercurrent transport), we would anticipate a high degree of sensitivity towards $t_{Pt,b}$ and $t_{Pt,t}$. This is indeed observed and is particularly noticeable for variation in $M_{R}$ vs $t_{Pt,b}$, where a strong dependence is found between $t_{Pt,b} =$ 0.6 and 2.5 nm samples. Returning now to $H_{c}$ values in Fig. 5, we find that structures possessing the same $t_{Co}$ exhibit comparable coercive fields. In the domain wall nucleation limited regime, i.e., for room temperature measurements at low $t_{Co}$ and moderate reversal times ($\sim$ 100 Oe/s field sweep rates), $H_{c}$ is expected to be predominantly dictated by $t_{Co}$ and Co microstructure^60,62^. While the invariance of $H_{c}$ may therefore be anticipated, given the constant $t_{Co}$ between samples, this also points to a consistent Co film microstructure across the differing Pt underlayer thicknesses and, potentially, similar fcc (111) texturing of the Co layer for all $t_{Pt}$^60^.

**Figure 6:**
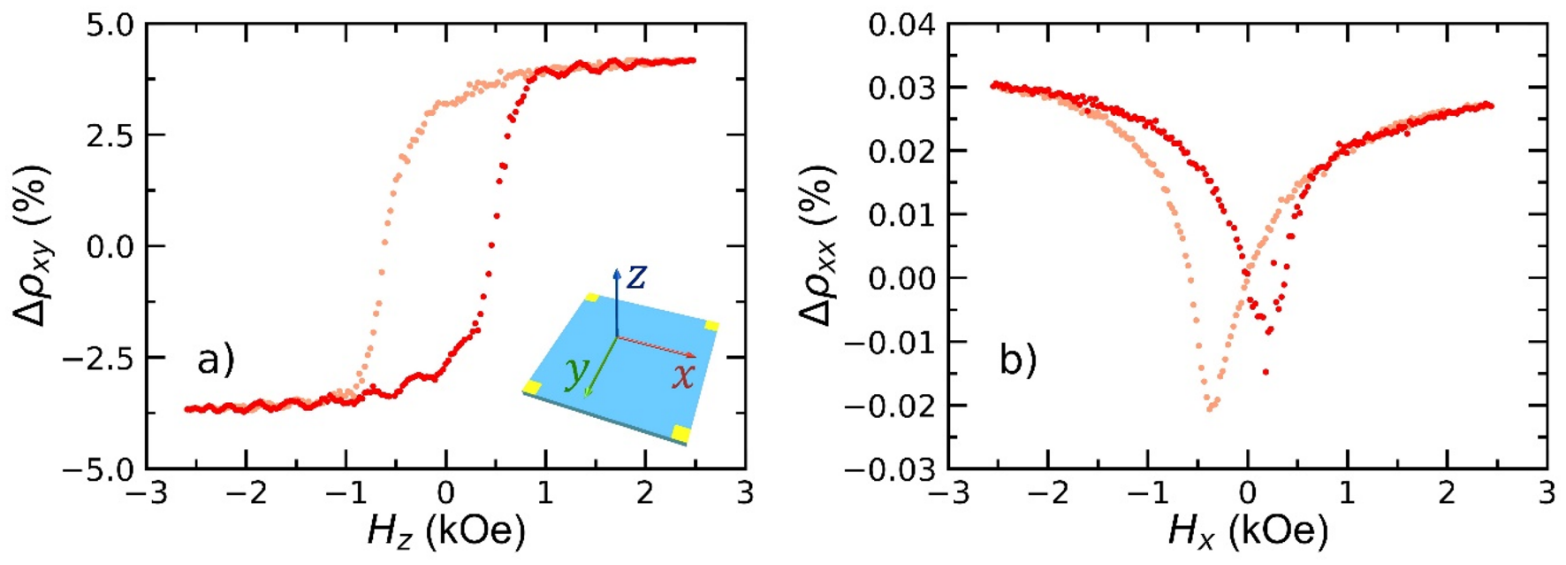
Magneto-transport characterisation of a Nb(15)/Pt(0.8)/Co(1)/Pt(0.6)/Cu(5)/MgO(3) multilayer. The sample is cooled down from room temperature in the absence of a magnetic field. Subsequent data is recorded at $T =$ 4 K. Degaussing takes place before each measurement. During measurements the field is swept at a rate of 25 Oe/s. Forward and reverse field sweeps are indicated by red and pink markers respectively. (**a**) Hall resistivity percentage change, ${\Delta }\rho_{xy}$, as a function of out-of-plane applied field, $H_{z}$. Note, ${\Delta }\rho_{xy} = \left( {\rho_{xy} \left( H \right) - \rho_{xy,0} } \right)/\rho_{xy,0}$, where $\rho_{xy,0} = \left[ {\rho_{forward} \left( {H = 0} \right) - \rho_{reverse} \left( {H = 0} \right)} \right]/2$. Inset, schematic of the measurement orientation. Yellow squares at the corners indicate electrical contact points. (**b**) AMR resistivity percentage change, ${\Delta }\rho_{xx}$, as a function of in-plane applied field, $H_{x}$. ${\Delta }\rho_{xx} = \left( {\rho_{xx} \left( H \right) - \rho_{xx} \left( {H = 0} \right)} \right)/\rho_{xx} \left( {H = 0} \right)$.

Finally, with a view to future superspintronic measurements, we consider magnetic reversal close to $T_{c}$. Magnetotransport measurements were performed at $T =$ 4 K ($T_{c} =$ 2.84 K) in both longitudinal, $\rho_{xx} = V_{x} \left( {H_{x} } \right){ }/I_{x}$ (i.e. $H_{x} \equiv H_{\parallel }$), and Hall geometries, $\rho_{xy} = V_{y} \left( {H_{z} } \right){ }/I_{x}$ (i.e. $H_{z} \equiv H_{ \bot }$), [where the subscript denotes the orientation axis, see Fig. 6a inset]. Figure 6a shows the normalised Hall resistance ${{ \Delta }}\rho_{xy} \left( {H_{z} } \right)$, which is sensitive to the anomalous Hall effect (AHE) in the Co layer. There we see an AHE loop consistent with out-of-plane easy axis reversal, in agreement with room temperature measurements. Comparison with MOKE data from Fig. 5 (green data) shows an increase in both $H_{c}$, from 108 to 534 Oe, and $M_{R} /M_{s}$, from 0.67 to 0.74. The increase in $M_{R}$ naturally points to an increase in PMA as $T$ decreases; this increase reflects the power law dependence for the interfacial anisotropy, $K_{s} \propto M_{s}^{\gamma } ($ with $\gamma = 3$ in Co^63^) and weak variation in $M_{s} \left( T \right)$ in Co thin films between 4 and 300 K (Curie temperature $T_{c}$ = 1400 K for bulk Co). Given domain wall nucleation is a thermally activated process, an increase in $H_{c}$ would naturally arise on cooling; the five-fold increase is, nonetheless, sizeable. This can be reconciled by the concomitant increase in $K_{s}$ and $M_{s}$ on decreasing $T$, which both act to increase the energy barrier to nucleation and reversal, thereby increasing $H_{c}$. For comparison, the normalised longitudinal magnetoresistance is shown in Fig. 6b, ${\Delta }\rho_{xx} \left( {H_{x} } \right)$, which is conventionally sensitive to the anisotropic magnetoresistance (AMR) of the Co layer. In contrast to Fig. 5a, the AMR signal shows signs of an *in-plane* easy axis reversal, which would potentially indicate a low anisotropy system, i.e. where $\mu _{0} {{M_s}^{2}} { \sim K_s}$, such that low remanence, low coercivity in-plane switching is found when the field is cycled in-plane, despite an overall out-of-plane easy axis^64,65^.

**Figure 7:**
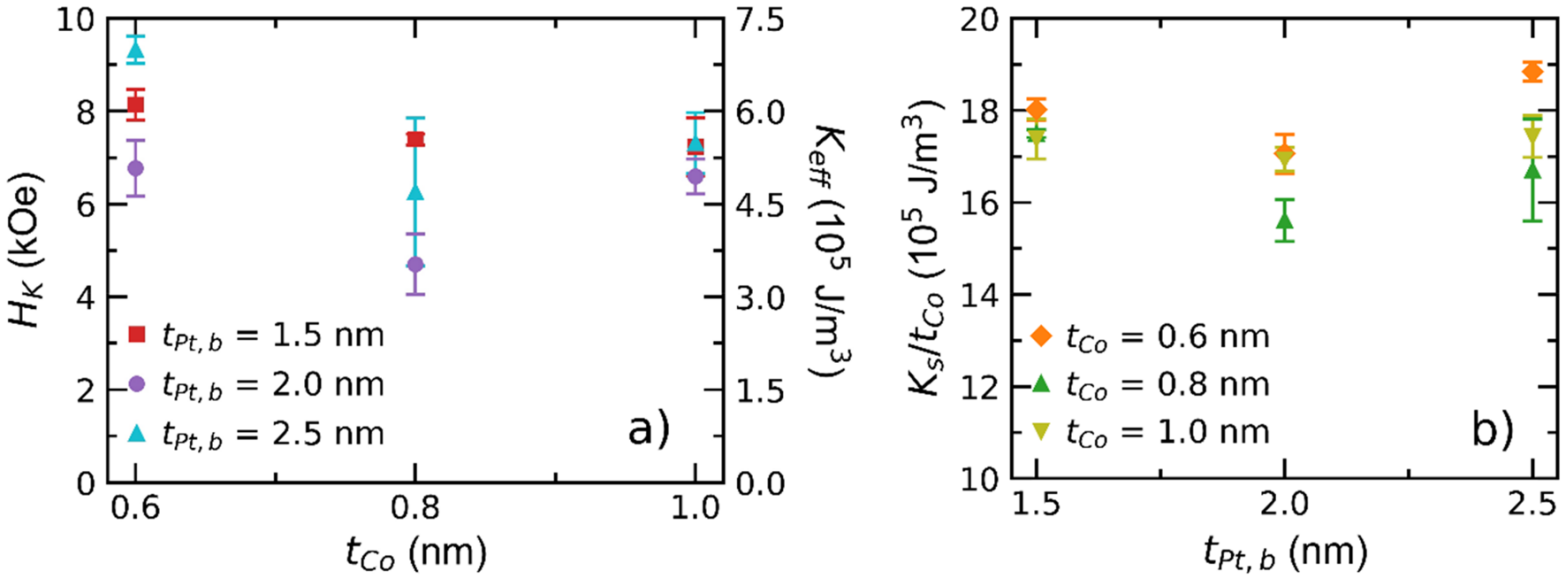
Magnetic characteristics Nb(15)/Pt($$t_{Pt,b}$$)/Co($$t_{Co} )$$/Pt(1.5)/MgO(3) samples, derived from magnetoresistance Hall measurements. Fields have been applied in-plane and swept at 200 Oe/s. (**a**) Perpendicular anisotropy field, $$H_{K}$$, (left axis) estimated from the hard-axis saturation field i.e. an in-plane applied field hall measurement, and the corresponding net anisotropy, $$K_{eff}$$, (right axis) as a function of cobalt thickness, $$t_{Co}$$, for each bottom Pt thickness. (**b**) Surface anisotropy per unit cobalt thickness, $$K_{s} /t_{Co}$$, as a function of bottom Pt thickness, for the same samples as in (**a**).

Further analysis of the interplay between $t_{Pt,b}$, $t_{Co}$ and the resulting effect on the Co anisotropy can be seen in Fig. 7. Here, the anisotropy field, $H_{K}$, i.e. the in-plane field required to saturate the sample, was measured, via the planar Hall effect, for samples with a constant $t_{Pt,t}$ = 1.5 nm and varying $t_{Pt,b}$ and $t_{Co}$. The measured anisotropy field is linked to the effective anisotropy constant, $K_{eff}$, by^66^

1 $$H_{K} = \frac{{2K_{eff} }}{{\mu_{0} M_{s} }}$$

where $M_{s}$ is assumed to be the bulk saturation magnetisation of Co: 1.4 $\times$ 10^6^ A/m. Figure 7a shows $H_{K}$ and the corresponding $K_{eff}$ vs $t_{Co}$, for various $t_{Pt,b}$. Here, 3 < $K_{eff}$ < 7.5 10^5^ J/m^3^ , which are in general agreement with studies of comparable Co and Pt thicknesses, using different refractory metal seed layers (such as Ru and Ta)^61,67,68^. No clear dependence of $K_{eff}$ on $t_{Pt,b}$ and $t_{Co}$ is found, which can most likely be attributed to the limited range of $t_{Pt,b}$ and $t_{Co}$ values explored. Considering both interfacial and shape anisotropy, the effective anisotropy is given by:

2 $$K_{eff} = \frac{{K_{s} }}{{t_{Co} }} - \frac{1}{2}\mu_{0} M_{s}^{2}$$

Here, $K_{s}$ denotes the interfacial anisotropy between the two Pt and Co interfaces and the second term represents the shape anistropy for the thin film. Figure 7b plots $K_{s} /t_{Co}$ vs $t_{Pt,b}$. Again, we see $K_{s} /t_{Co}$ to take an almost constant value of $\sim$ 17.5 $\times$ 10^5^ J/m^3^, which is comparable to $K_{s} /t_{Co} =$ 18 $\times$ 10^5^ J/m^3^ seen for Ta buffered Pt/Co/Pt in Ref.^67^, again reflecting the substantial PMA generated for the buffered films. This value of $K_{eff}$ indicates a system close to the spin reorientation transition^65^ and, as such, naturally explain the apparent easy axis switching seen in both in-plane (Fig. 6a) and out-of-plane (Fig. 6b) magnetotransport.

## Conclusion

In this work we have investigated the superconducting and magnetic properties of EBE polycrystalline Nb/Pt/Co/Pt heterostructures. We find EBE provides a facile route to high quality Nb thin films, here demonstrating reliable $T_{c}$ values exceeding 5 K, even down to $t_{Nb} =$ 5 nm in unannealed, polycrystalline films. Furthermore, we find that Al capped Nb films above 5 nm are amenable to post-growth annealing, with increases in $T_{c}$ of over 2 K consistently achieved. The ability to deposit thin *S-F* heterostructures via line-of-sight deposition methods opens several possibilities for future superspintronic applications. First, the study of thin Nb layers offers the potential for insights into crossed Andreev reflection, elastic co-tunnelling and induced triplet pairing states of triplet supercurrents^69,70^, as well as enhancing interface effects in fundamental spin transport studies. Furthermore, the ability to fabricate sub-10 nm thick superconducting films also allows for simpler integration of *S* layers into more complex device geometries, including thin superconducting spin valve devices. As EBE is a line-of-sight deposition technique, it has the further advantages of being well suited to (positive) mask-based lithography, glancing angle deposition, and templated coating, which avoids the need for reductive etching and readily allows for 3D superspintronic structures.

By exploring the properties of EBE Pt/Co/Pt trilayers, with and without Nb(15) underlayers, we demonstrate that Nb acts as an effective buffer layer for Pt growth, yielding significant PMA in ultrathin ($t_{Pt,b}$ and $t_{Pt,t} <$ 1 nm) heterostructures. Out-of-plane remanence in Co thin films is demonstrated both at room temperature, using MOKE microscopy, and at low temperature, as evidenced via magnetotransport measurements above $T_{c}$.

The results emphasise the potential for all-EBE heterostructures to provide new, relatively simple routes to developing superspintronic devices where ultrathin layers with mixed magnetic anisotropy are key, and potentially paves a way to unravelling the intricate physics underpinning the interfacial spin transport mechanisms active in *S-F* structures.

## Methods

The presented samples were fabricated via EBE in a multi-source UHV deposition system. Multilayers were deposited in the same vacuum step, sequentially, to avoid interface contamination and oxidation. The growth chamber is pumped using an ion pump and liquid nitrogen cryoshroud, with base pressure of order 10^–10^ mBar. During growth, a residual pressure of the order 10^–8^ mBar was maintained. It is known that O readily reacts with Nb and that interstitial O has a notable impact on transition temperature, decreasing ${T}_{c}$ by 0.93 K per at.%^71^. Residual gas analysis, performed immediately before and after Nb deposition, reported levels of O below the detection threshold ($\lesssim$ 10^–11^ mBar), with the chamber residual pressure dominated by H_2_ due to the low pumping efficiency of the molecule via ion pump. The Nb hearth has a large source-substrate distance of 725 mm, providing highly uniform film growth.

All samples were grown on thermally oxidised Si (100) substrates (SiO_x_ thickness = 200 nm) seated on an unheated sample stage. Source material purities and deposition rates were: Nb, 99.95%, 0.3 Å/s; Pt, 99.99%, 0.05 Å/s; Co, 99.95%, 0.05 Å/s; Cu, 99.999%, 0.3 Å/s; MgO, 99.95%, 0.25 Å/s; and Al, 99.999%, 0.2 Å/s. Deposited thicknesses were monitored during growth using a calibrated quartz crystal monitors and verified, post-deposition, using grazing incidence XRR. Samples are annealed *ex-situ* on a plate heater at a base vacuum of approximately 10^–6^ mbar and temperatures of either 300, 400, 500, 600 °C for 1 h (not including the time taken to ramp up to and down from these temperatures). A cryoshroud and turbo pump are used to maintain HV conditions during annealing.

Post fabrication, XRD and XRR analysis were performed using a Rigaku Smartlab X-ray diffractometer, with a copper anode (incident radiation = Cu K $\alpha_{1}$). Temperature- and magnetic field-dependent electrical transport measurements were carried out in a closed cycle ^4^He cryostat with an externally applied magnetic field, up to 10 kOe. Samples were measured either in the van der Pauw geometry^72^ or the traditional in-line 4-point-probe transport geometry using a Lakeshore 372 AC Resistance bridge set to a constant current mode, with contacts permutated via a Keithley 3706A-S system switchboard. All devices which are cooled are done so in zero applied field and degaussed before measurements performed. Superconducting transition temperatures of each sample were measured for multiple warmups in order to calculate the statistical error. This was typically found to be ~ 20 mK. Room temperature magneto-optical Kerr effect and SQUID VSM measurements were performed using a Durham Magneto Optics Ltd NanoMOKE3 and Quantum Design MPMS 3 magnetometer respectively. Microstructural characterisation was performed using an Agilent 5600LS atomic force microscope (AFM). XPS data was acquired with a Phi Versa Probe III using a monochromated Al K_α_ source ($h\nu$ = 1486.6 eV) at a pressure of 10^–10^ mbar. The spectrometer resolution was determined by measuring an Ar^+^ ion sputtered polycrystalline Au foil and fitting the Fermi level using a Fermi–Dirac distribution function convoluted with a Gaussian. The full width at half maximum was determined to be 0.5 eV. As the samples were deposited on a non-conducting substrate, charge neutralisation was performed using an electron flood gun (compensating charging) coupled with a low-energy Argon ion source (to compensate from saturation of electrons from the electron gun). Core levels were measured with an analyser pass energy of 55 eV and a step size of 0.01 eV. With regards to analysing such spectra Voigt functions were employed for the insulating Al_2_O_3_, MgO, and NbO_x_ core levels. However, for metallic Nb peaks, a Doniach Šunjić lineshape was used to account for the high binding energy tailing resulting from energy loss of the photoelectrons to conduction band plasmons.

## Data Availability

The datasets generated during and/or analysed during the current study are available from the corresponding author on reasonable request.
